# Variations in the Branching Pattern and Course of the Left Axillary Artery: A Cadaveric Case Report

**DOI:** 10.7759/cureus.40852

**Published:** 2023-06-23

**Authors:** Palak P Oza, Halley C Yung, Kiran Vaz Grace

**Affiliations:** 1 Department of Molecular, Cellular and Biomedical Sciences, City University of New York (CUNY) School of Medicine, New York, USA

**Keywords:** anteromedial course, common trunk, branching variation, brachial plexus, axillary artery

## Abstract

The axillary artery is the primary arterial supply of the upper limb and functions as a key landmark in the region of the axilla. Anatomical variations in the axillary artery may arise from abnormal angiogenesis in the upper limb bud during embryological development. The routine dissection of the upper limb of an 88-year-old male cadaver revealed unilateral variations in the left axillary artery, including an abnormal anteromedial course with respect to the divisions and cords of the brachial plexus, while no variations were observed in the right axillary artery. Variations in branching were observed in each part of the artery. In the first part of the artery, variations included an accessory branch coursing towards the clavicle and another to the subscapularis muscle. A total of four branches arose from the second part of the artery: a thoracoacromial artery, an accessory pectoral branch, and two common trunks. The first common trunk gave off the alar thoracic artery, an accessory lateral thoracic artery, and the subscapular artery, which further gave off the thoracodorsal and lateral thoracic arteries, prior to continuing as the circumflex scapular artery. The second common trunk yielded the anterior and posterior circumflex humeral arteries prior to continuing as the profunda brachii artery. No branches arose from the third part of the artery. Awareness of variations in the course and branching patterns of the axillary artery as observed in this cadaveric donor is essential for anesthetic, radiographic, surgical, and other interventional procedures of the upper limb.

## Introduction

The axillary artery (AA) is a continuation of the subclavian artery, commencing at the lateral border of the first rib and extending to the inferior border of the teres major muscle, after which it continues as the brachial artery [1]. The artery is divided into three sections by its relationship to the pectoralis minor muscle lying anterior to it. The first part of the AA extends from the lateral border of the first rib to the medial border of the pectoralis minor muscle and gives rise to the superior thoracic artery. The second part of the AA lies deep to the pectoralis minor muscle and gives off two branches: the thoracoacromial artery and the lateral thoracic artery, the former of which further divides to give off pectoral, clavicular, acromial, and deltoid branches. The third part of the AA extends from the lateral border of the pectoralis minor muscle to the inferior border of the teres major muscle and gives off three branches. The subscapular artery is the largest branch of this segment, and it branches into the thoracodorsal artery and circumflex scapular artery. The other two branches are the anterior and posterior circumflex humeral arteries, which arise approximately opposite the origin of the subscapular artery and course around the surgical neck of the humerus, where they anastomose [1]. 

The course of the AA is closely related to the brachial plexus, making it a landmark in clinical procedures within the region of the axilla. The first part of the AA courses anterior to the brachial plexus, then passes between the cords of the brachial plexus so that the second part of the AA is bordered laterally by the lateral cord, medially by the medial cord, and posteriorly by the posterior cord, after which the median nerve can be seen anterior to the artery [2].

Variations in the branching patterns of the AA and its positional relationship to the brachial plexus are of importance in the context of interventions including brachial plexus blocks, surgical repair of trauma to the region, and other invasive procedures within the axilla. Awareness of these variations may reduce the risk of complications during such procedures. Numerous variations in the branching patterns of the AA have been previously described, but an aberrant positional relationship with the brachial plexus has been infrequently reported. Here, we report extensive branching variations within the AA as well as a deviant positional relationship between the AA and the brachial plexus. 

## Case presentation

**Figure 1:**
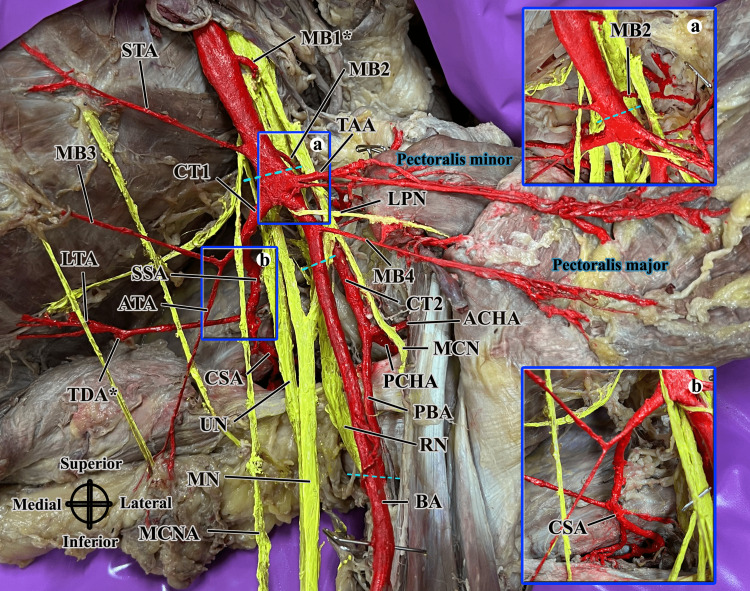
Branching variations and anteromedial course of the left AA in relation to the brachial plexus. Asterisks (*) indicate branches that were severed during the dissection. Nerve structures are painted yellow, arterial structures painted red, muscles labeled in blue font, and dashed blue lines indicate the boundaries of the parts of the AA. Directional indication is provided in the lower left corner. (a) A close-up of MB2, an accessory branch to the subscapularis muscle. (b) A close-up of the CSA. AA: axillary artery; ACHA: anterior circumflex humeral artery; ATA: alar thoracic artery; BA: brachial artery; CSA: circumflex scapular artery; CT1; common trunk 1; CT 2: common trunk 2; LPN: lateral pectoral nerve; LTA: lateral thoracic artery; MB1: muscular branch 1, seen coursing to the clavicular region; MB2: muscular branch 2, seen piercing the subscapularis muscle; MB3: muscular branch 3, an accessory lateral thoracic branch; MB4: muscular branch 4, an accessory pectoral branch; MCN: musculocutaneous nerve; MCNA: medial cutaneous nerve of the arm; MN: median nerve; PBA: profunda brachii artery; PCHA: posterior circumflex humeral artery; RN: radial nerve; SSA: subscapular artery; STA: superior thoracic artery; TAA: thoracoacromial artery; TDA: thoracodorsal artery; UN: ulnar nerve.

**Figure 2:**
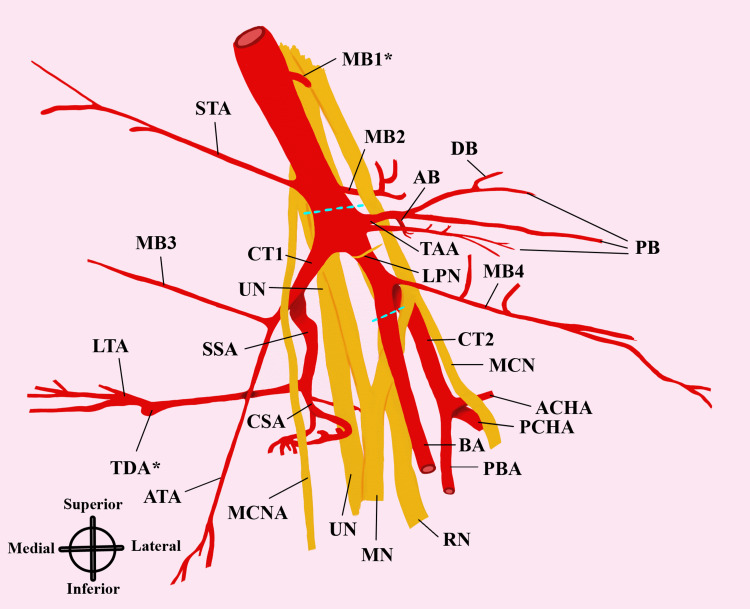
A schematic representation of the observed AA variations. Asterisks (*) indicate branches that were severed during the dissection. Nerve structures are in yellow, arterial structures in red, and dashed blue lines indicate the boundaries of the parts of the AA. AA: axillary artery; AB: acromial branch of thoracoacromial artery; ACHA: anterior circumflex humeral artery; ATA: alar thoracic artery; BA: brachial artery; CSA: circumflex scapular artery; CT1: common trunk 1; CT2: common trunk 2; DB: deltoid branch of the thoracoacromial artery; LPN: lateral pectoral nerve; LTA: lateral thoracic artery; MB1: muscular branch 1, seen coursing to the clavicular region; MB2: muscular branch 2, seen piercing the subscapularis muscle; MB3: muscular branch 3, an accessory thoracic branch; MB4: muscular branch 4, an accessory pectoral branch; MCN: musculocutaneous nerve; MCNA: medial cutaneous nerve of the arm; MN: median nerve; PB: pectoral branch of the thoracoacromial artery; PBA: profunda brachii artery; PCHA: posterior circumflex humeral artery; RN: radial nerve; SSA: subscapular artery; STA: superior thoracic artery; TAA: thoracoacromial artery; TDA: thoracodorsal artery; UN: ulnar nerve.

The routine dissection of the left upper limb of an 88-year-old male donor revealed several variations in the branching patterns of the AA and its positional relationship to the brachial plexus (Figures 1, 2). Briefly, the AA was exposed along with the axillary vein and brachial plexus after reflection of the skin in the axilla, followed by reflection of the pectoralis major and minor muscles. Arterial structures were painted red and nerve structures were painted yellow using acrylic paint for better visualization. The observed variations in the course of the artery and its branching patterns are reported here. 

All three parts of the AA, as well as the accompanying axillary vein, demonstrated an abnormal anteromedial course with respect to all cords of the brachial plexus. Marked variations from the normal branching pattern were observed in all three parts of the artery. 

The first part of the AA gave rise to three branches in total. The superior thoracic artery originated on the medial aspect of the AA, slightly more distal to its normal origin, and crossed anterior to all components of the brachial plexus to supply the superior thoracic wall. Two anomalous branches were noted on the lateral aspect of the first part of the AA: MB1, a thick branch that was severed during the course of the dissection, was seen originating on the lateral aspect of the AA proximal to the origin of the superior thoracic artery and observed coursing superolaterally to the clavicular region. The second anomalous branch, MB2, originated laterally at the branching level of the superior thoracic artery and was observed coursing deep to the lateral cord, superficial to the posterior cord, and terminating as multiple smaller branches piercing the subscapularis muscle (Figure 1a). 

Four branches, including two identified as common trunks, were given off from the second part of the AA. The thoracoacromial artery originated on the lateral aspect of the AA, yielding three pectoral branches, one deltoid branch, and one acromial branch (Figure 2); no clavicular branch was observed. At the same level of origin of the thoracoacromial artery, the first common trunk (CT1) was observed from the medial aspect of the AA. CT1 gave off (1) a branch that further divided into a muscular branch piercing the serratus anterior muscle, identified as an accessory lateral thoracic artery, and another cutaneous branch that coursed distally to supply the skin, subcutaneous fat, and axillary lymph nodes, which we identified as the alar thoracic artery based on previous reports of the same [3,4]; (2) the second branch of CT1 was the subscapular artery, which further divided into the circumflex scapular artery (Figure 1b) and the lateral thoracic artery.

The lateral thoracic artery gave rise to the thoracodorsal artery, which was severed during the course of dissection. Distal to the origins of the thoracoacromial artery and CT1, an accessory branch to the pectoralis major and minor muscles, referred to as MB4, was observed branching from the lateral aspect of the AA and lying at the level of the branching of the musculocutaneous nerve and lateral root of the median nerve from the lateral cord of the brachial plexus. The second common trunk (CT2) arose from the posterolateral aspect of the second part of the AA, at the level of branching of the musculocutaneous nerve and the lateral root of the median nerve from the lateral cord of the brachial plexus. CT2 coursed deep to both nerve branches and gave off the anterior circumflex humeral artery and posterior circumflex humeral artery proximal to the tendon of the teres minor muscle, prior to continuing as the profunda brachii artery. No variations were observed in the course or branching of the brachial artery, which continued to divide into the radial and ulnar arteries at the cubital fossa as expected. No branches were seen arising from the third part of the AA.

## Discussion

Knowledge of variations within the AA is essential for clinicians in the context of radiographic, anesthetic, and surgical procedures. A range of branching variations of the AA have been previously described, including reports of a common origin of the lateral thoracic and subscapular arteries [1], absence of the thoracoacromial artery with all of its branches originating directly from the AA [5], anastomosis of the posterior circumflex with intercostal arteries [6], and origin of the lateral thoracic artery from the third part of the AA [7]. 

One previously described branching variation in the AA is the presence of a common trunk that yields several major branches that classically originate at separate locations on the artery [5,6]. In this case, we report two common trunks, both arising from the second part of the AA. The proximal common trunk, CT1, gave rise to a lateral thoracic, alar thoracic, and subscapular artery that gave off an accessory lateral thoracic artery. The alar thoracic artery, which has been described in previous reports [3,4], continued distally to supply the skin and fat of the axilla. The distal common trunk, CT2, gave off the anterior and posterior circumflex humeral arteries and continued as the profunda brachii; a similar branching pattern, but with an additional subscapular branch, has been previously described from the third part of the AA [8,9]. Single common trunks arising from the second [10,11] and third [5,6,8,12] parts of the AA have been described; however, to our knowledge, the presence of two separate common trunks both arising from the second part of the AA was previously undocumented. 

Branching variations within the AA are important to consider for several reasons. Firstly, the passage of nerves through this region is inextricably linked to the location and course of the arterial branches. Additionally, the use of AA branches for microvascular grafts [13] and procedures such as AA reconstruction, revascularization using bypass procedures involving the AA, and reconstructive surgeries using muscle flaps from the region necessitate awareness of potential branching variations to prevent unnecessary complications.

An additional point of interest is the variation in the positional relationship between the AA and brachial plexus. Traditionally, the first part of the AA courses anterior to the brachial plexus prior to passing between the medial and lateral roots of the median nerve; the second part of the artery is then surrounded laterally, medially, and posteriorly by the corresponding cords of the brachial plexus. Nerve roots from the lateral and medial cords join to form the median nerve, which courses anterior to the artery. Deviations from this relationship have been less extensively reported than those in arterial branching patterns. 

Variations in the course of the AA may be attributed to deviations from normal embryological development. The left AA is derived from the left seventh cervical intersegmental artery, which arises from the dorsal aorta. The dorsal aortae give rise to a set of seven cervical intersegmental arteries bilaterally, of which all but the seventh merge and develop into the vertebral artery, while the seventh develops into the subclavian artery, which continues as the AA. Variations in the course of the AA can be seen in cases where an intersegmental artery at a different level, such as the sixth, eighth, or ninth intersegmental arteries, gives rise to the AA [14].

We report here an anteromedial course of the AA in relation to the cords of the brachial plexus. One case series reported an aberrant positional relationship between the AA and the brachial plexus in 2.0% of cases [15], comparable to the 2.3% prevalence reported in a separate study [14]. Of these, the most common variant reported was an anteromedial course of the AA in relation to the brachial plexus [14,15]. This particular variant, which is also present in the current case, is suggested to result from the formation of the AA from the ninth intersegmental artery during development [14]. Other reports include a medial [16,17] and deep [18,2] course of the AA in relation to the cords of the brachial plexus. Often, these positional variations are observed alongside deviations in the branching patterns of the brachial plexus, raising the question of whether the aberrant development of the brachial plexus branching pattern is responsible for the atypical development of the AA or vice versa [14]. Importantly, no brachial plexus anomalies were noted in the current case. 

The variation in the positional relationship of the neurovascular structures within the axilla is of tremendous importance when considering invasive procedures in the upper limb. For instance, in the use of an axillary approach to a brachial plexus block performed for regional anesthesia of the upper limb, the AA is used as a landmark [19,20]; thus, the risks of incomplete block and intravascular injection may be increased in patients with variations in the expected relationship between the artery and the plexus.

## Conclusions

In this case, we report an anteromedial course of the left AA to the brachial plexus and several branching variations observed unilaterally. The AA gave off a total of seven branches, three of which were accessory muscular branches to the clavicular region, the subscapularis muscle, and the pectoralis major and minor muscles, and two of which were common trunks seen arising from the second part of the AA. The lateral thoracic and subscapular arteries were derived from the proximal common trunk, CT1, alongside an accessory lateral thoracic artery and the alar thoracic artery. The distal common trunk, CT2, gave off the anterior and posterior circumflex humeral arteries prior to continuing as the profunda brachii artery. No branches were seen from the third part of the AA. The presence of two separate common trunks both originating from the second part of the AA was, to our knowledge, previously undocumented. In the clinical context, such variations, when unexpected, may introduce a risk of complications during anesthetic and surgical procedures. Awareness of the variations in the branching and course of the vasculature of the axilla is thus of significance in imaging and interventional procedures in this region. 

## References

[1] Yang K, Lee H, Choi IJ, Jeong W, Kim HT, Wei Q, Lee JH (2021). Topography and anatomical variations of the axillary artery. Biomed Res Int.

[2] Honma S, Kawai K, Koizumi M, Kodama K (2003). Aberrant axillary artery descending deep beneath the brachial plexus. Anat Sci Int.

[3] Rustagi SM, Sharma M, Singh N, Mehta V, Suri RK, Rath G (2015). Peripheral communications of intercostobrachial nerve peripheral communications of the intercostobrachial nerve in relation to the alar thoracic artery. Adv Biomed Res.

[4] Suman T, Afroze MKH (2020). Anatomical study of variations in the origin of axillary artery branches and its clinical emphasis. Acad Anat Int.

[5] Astik R, Dave U (2012). Variations in branching pattern of the axillary artery: a study in 40 human cadavers. J Vasc Bras.

[6] Banerjee A, Kumari C, Jhajhria SK (2017). Variation in the branching pattern of third part of axillary artery- a case report. J Clin Diagn Res.

[7] Mathis M, Marshall J, Hammer L, Chambers P, Rosario MG (2018). Anatomical variations of the axillary artery of human cadavers. FASEB Journal.

[8] Aastha Aastha, Jain A, Kumar MS (2015). An unusual variation of axillary artery: a case report. J Clin Diagn Res.

[9] Ramesh Rao T, Shetty P, Suresh R (2008). Abnormal branching pattern of the axillary artery and its clinical significance. Int J Morphol.

[10] Bhat KM, Gowda S, Potu BK, Rao MS (2008). A unique branching pattern of the axillary artery in a South Indian male cadaver. Bratisl Lek Listy.

[11] Shantakumar SR, Mohandas Rao KG (2012). Variant branching pattern of axillary artery: a case report. Case Rep Vasc Med.

[12] Bagoji IB, Hadimani GA, Bannur BM, Patil BG, Bharatha A (2013). A unique branching pattern of the axillary artery: a case report. J Clin Diagn Res.

[13] Valnicek SM, Mosher M, Hopkins JK, Rockwell WB (2004). The subscapular arterial tree as a source of microvascular arterial grafts. Plast Reconstr Surg.

[14] Yang HJ, Gil YC, Lee HY (2009). Intersegmental origin of the axillary artery and accompanying variation in the brachial plexus. Clin Anat.

[15] Pandey SK, Shukla VK (2007). Anatomical variations of the cords of brachial plexus and the median nerve. Clin Anat.

[16] Jamuna M (2011). Clinically significant variations of the cords of the brachial plexus in relation to axillary artery. Int J Anat Var.

[17] Satyanarayana N, Vishwakarma N, Kumar GP, Guha R, Datta AK, Sunitha P (2009). Variation in relation of cords of brachial plexus and their branches with axillary and brachial arteries--a case report. Nepal Med Coll J.

[18] Aggarwal A, Puri N, Aggarwal AK, Harjeet K, Sahni D (2010). Anatomical variation in formation of brachial plexus and its branching. Surg Radiol Anat.

[19] Vazin M, Jensen K, Kristensen DL (2016). Low-volume brachial plexus block providing surgical anesthesia for distal arm surgery comparing supraclavicular, infraclavicular, and axillary approach: a randomized observer blind trial. Biomed Res Int.

[20] Song IA, Gil NS, Choi EY, Sim SE, Min SW, Ro YJ, Kim CS (2011). Axillary approach versus the infraclavicular approach in ultrasound-guided brachial plexus block: comparison of anesthetic time. Korean J Anesthesiol.

